# Gene expression and the evolution of phenotypic diversity in social wasps

**DOI:** 10.1186/1741-7007-5-23

**Published:** 2007-05-15

**Authors:** Eric A Hoffman, Michael AD Goodisman

**Affiliations:** 1 Department of Biology, University of Central Florida, 4000 Central Florida Blvd, Orlando, FL 32816, USA; 2 School of Biology, Georgia Institute of Technology, 310 Ferst Drive, Atlanta, GA 30332, USA

## Abstract

**Background:**

Organisms are capable of developing different phenotypes by altering the genes they express. This phenotypic plasticity provides a means for species to respond effectively to environmental conditions. One of the most dramatic examples of phenotypic plasticity occurs in the highly social hymenopteran insects (ants, social bees, and social wasps), where distinct castes and sexes all arise from the same genes. To elucidate how variation in patterns of gene expression affects phenotypic variation, we conducted a study to simultaneously address the influence of developmental stage, sex, and caste on patterns of gene expression in *Vespula *wasps. Furthermore, we compared the patterns found in this species to those found in other taxa in order to investigate how variation in gene expression leads to phenotypic evolution.

**Results:**

We constructed 11 different cDNA libraries derived from various developmental stages and castes of *Vespula squamosa*. Comparisons of overall expression patterns indicated that gene-expression differences distinguishing developmental stages were greater than expression differences differentiating sex or caste. Furthermore, we determined that certain sets of genes showed similar patterns of expression in the same phenotypic forms of different species. Specifically, larvae upregulated genes related to metabolism and genes possessing structural activity. Surprisingly, our data indicated that at least a few specific gene functions and at least one specific gene family are important components of caste differentiation across social insect taxa.

**Conclusion:**

Despite research on various aspects of development originating from model systems, growth in understanding how development is related to phenotypic diversity relies on a growing literature of contrasting studies in non-model systems. In this study, we found that comparisons of patterns of gene expression with model systems highlighted areas of conserved and convergent developmental evolution across diverse taxa. Indeed, conserved biological functions across species implicated key functions related to how phenotypes are built. Finally, overall differences between social insect taxa suggest that the independent evolution of caste arose via distinct developmental trajectories.

## Background

A fundamental goal of the burgeoning field of evolutionary developmental biology is to understand how differences in gene expression contribute to phenotypic diversity. Phenotypic plasticity, the ability of a single genotype to produce alternate forms of morphology, physiology or behavior in response to environmental conditions [1-3], provides a unique opportunity to investigate environmental influence on gene expression. Phenotypic plasticity is taxonomically widespread and usually results in continuous phenotypic variation [2,4]. However, some organisms exhibit phenotypic plasticity such that two or more discrete alternative phenotypes (without intermediate forms) are produced. This type of variation is called a polyphenism [5]. Because the phenotypic differences that exist among morphs can arise from an identical genome, polyphenisms provide an ideal means to explore how differential gene expression drives phenotypic diversity [6].

Highly social hymenopteran insects (ants, social bees, and social wasps) present one of the most striking examples of polyphenism. Hymenopteran queens, workers, and males all possess the same genes (although females are diploid and males are haploid), unlike many other animals, where sex chromosomes play a role in sex determination. Therefore, the phenotypic differences among hymenopteran social insect castes, as well as sexes, are derived from variation in gene expression.

**Figure 1:**
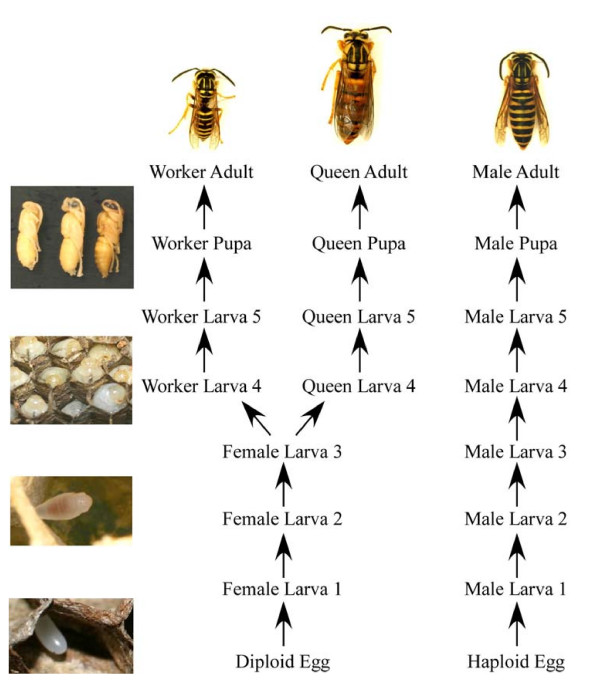
*V. squamosa *developmental pathways and stages. Haploid eggs develop into males. Diploid eggs give rise to workers and queens.

In this study, we investigated the molecular underpinnings involved in the development of the social wasp *Vespula squamosa*. *Vespula *wasps are a particularly good taxon in which to study phenotypic evolution, for several reasons. First, *Vespula *wasps display distinct female castes; queens differ from workers in size, color, behavior, body proportions, and physiology [7,8] (Figure 1). Second, *Vespula *wasps display remarkable similarities to *Apis *bees, although the two taxa are only distantly related [9]. Moreover, the complex caste and social systems found in the two taxa arose via independent evolutionary events. This point is of fundamental importance, because comparative analysis of development in *Vespula *and *Apis *will reveal if analogous environmentally induced phenotypes are generated through similar patterns of gene expression. Finally, *Vespula *queens and workers are reared in distinct cells; this key feature allows the developmental fate of larvae to be known very early in ontogeny [7].

This study addressed the following three important questions related to the evolution and development of queens, workers and males in *Vespula *wasps:

(i) How do patterns of gene expression differ among developmentally distinct phenotypes?

(ii) Are sex-specific developmental patterns similar across insect species?

(iii) Do caste-specific developmental patterns display convergent evolution?

## Results and discussion

### Expression patterns and developmental stage

**Figure 2:**
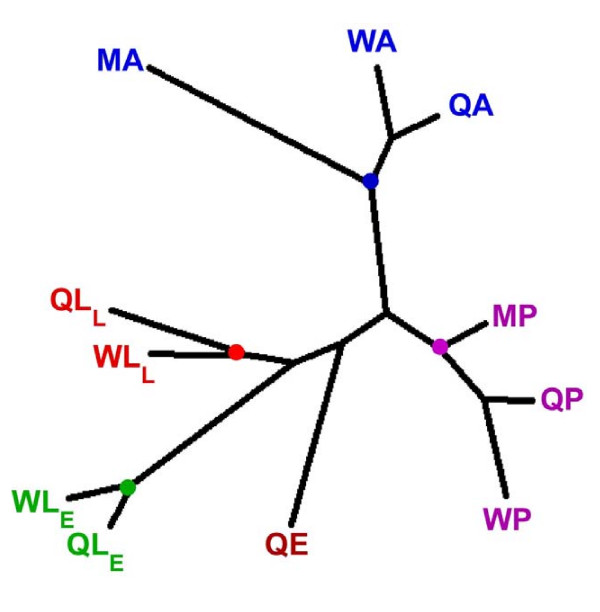
Relationships among *V. squamosa *life stages based upon EST frequencies. Colored dots indicate where libraries derived from developmentally similar stages cluster. Q, queen; W, worker; M, male; E, egg; L_E_, early larval instars; L_L_, late larval instars; P, pupa; A, adult.

The most striking result from our data is that developmental stage (i.e., larva, pupa, and adult) plays a much larger role in establishing patterns of gene expression than either caste or sex (Figure 2). In fact, developmental time is the critical factor in grouping the libraries by overall expression pattern. Thus, individuals of the same developmental age express many genes in common regardless of their caste or sex.

Which genes contribute to the differences among *V. squamosa *libraries and thus provided insight into the molecular processes associated with development in this taxon? A general χ^2 ^test (significance threshold at *p *< 0.01; [10]) identified 52 genes that were differentially expressed among libraries (Table 1). For example, hexamerin-like storage proteins (VSQ019, VSQ232, VSQ233, VSQ292; Table 1), which have been implicated in the development of other insect species (see below), also showed a distinctive pattern of upregulation during the late larval stages of *V. squamosa*. Additionally, the expressed sequence tags (ESTs) VSQ318 and VSQ031 are both members of the odorant-binding protein family (Table 1), which exhibit distinctive patterns of expression, falling along developmental lines. Interestingly, differentially expressed odorant-binding proteins have been implicated as key regulators of social behavior in other social insects [11].

Two other genes show patterns of expression similar to those observed in other species. First, VSQ445, which is homologous to the German cockroach major allergen Bla g 1, is upregulated in adult females, as is the case in cockroaches [12]. Second, VSQ709, which is upregulated in queen eggs, most closely matches the *Pisum sativum *putative senescence-associated protein. Surprisingly, Sharf et al [13] found that the same gene homolog was upregulated in immature reproductives of the termite *Reticulitermes flavipes*. They suggest that this gene may play a role in ribosomal filtering [14], owing to the similarity of *R. flavipes *(and VSQ709) transcript to 28s rRNA-like sequences.

Finally, several genes of unknown function or those with low or no known homology also showed characteristic expression patterns at particular developmental stages. For example, both VSQ056 and VSQ058 were upregulated during the early and late larval developmental stages, whereas VSQ943 exhibits a striking pattern of upregulation during the female pupal stages (Table 1). The role of these genes is currently unknown, but their expression patterns indicate that further research into their functions is warranted.

The general expression patterns found in *V. squamosa *support previous studies that have investigated gene-expression patterns among developmental stages. For example, Mathavan et al [15] found clearly demarcated transcript clusters in five different developmental stages during embryogenesis of the zebrafish. Wagner et al [16] also found an orderly progression in transcription through time during embryogenesis of the mouse. Working in a taxon more closely related to wasps, Arbeitman et al [17] discovered that the major breaks in gene-expression clustering of *Drosophila melanogaster *occurred between life stages. Similar to our results, Arbeitman et al found that expression patterns for both male and female adults grouped closely together despite the apparent morphological, physiological and behavioral differences that exist between sexes. In contrast to our results, they found that larval expression was more similar to that of adults, whereas expression of embryos was more similar to that of pupae. The *V. squamosa *data suggest a more temporal pattern, with EST frequencies more similar between adults and pupae than between adults and larvae.

To investigate patterns of gene expression in *V. squamosa *further, we used information from the gene ontology (GO) classifications [18]. Use of GO classifications enabled us to determine whether there were conserved biological functions across species that demonstrated how phenotypes were built. In general, gene function may be remarkably conserved, with broad temporal patterns in gene class utilization showing similar patterns through mouse embryogenesis and development of *D. melanogaster *[19]. To determine the extent to which conserved biological functions persist across species, we searched for GO similarities to other insects.

**Figure 3:**
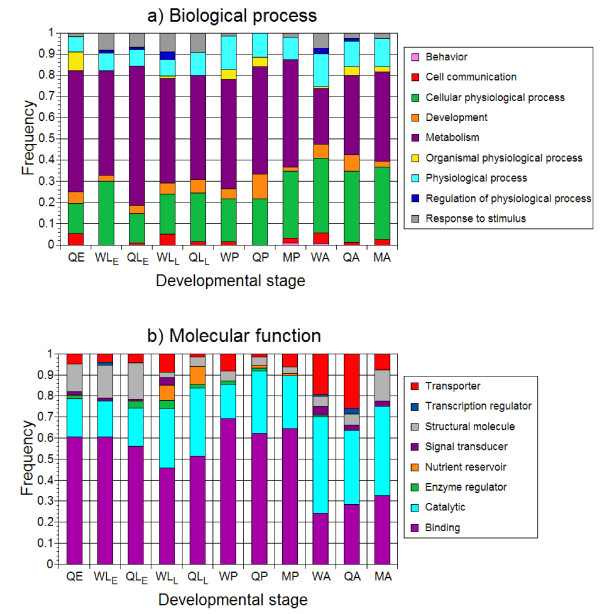
Proportion of transcripts from 11 cDNA libraries falling into different categories for **(A) **biological processes and **(B) **molecular function gene ontology. Q, queen; W, worker; M, male; E, egg; L_E_, early larval instars; L_L_, late larval instars; P, pupa; A, adult.

Goodisman et al [20] compared gene function similarities between *Camponotus festinatus *ants and *D. melanogaster*, and found that some patterns persisted between these species. In particular, the larvae of both species upregulated genes that were involved in protein production and possessed structural activity relative to those in adults. For both of these GO functions, the patterns hold true in *V. squamosa *when ESTs that show significant similarity to *D. melanogaster *genes are considered. The mean ± SE number of transcripts associated with protein metabolism is 51.5 ± 15.5 in larvae and 25.3 ± 4.8 in adults, and the mean ± SE number of transcripts possessing structural activity is 16.5 ± 4.5 in larvae and 5.3 ± 0.7 in adults (Figure 3). As with other studies, our results suggest that genes expressed throughout immature stages of holometabolous insects are associated with growth.

### Expression patterns and sex differences

Sex influences patterns of gene expression in *V. squamosa*. Within both branches of the neighbor-joining tree that contain both sexes (pupal and adult), the male patterns of gene expression are more different and hence diverge before the two female castes (Figure 2). Thus, despite the dramatic phenotypic differences between queens and workers, they are still more similar to each other in terms of gene expression than either is to males. This result is consistent with studies in *Caenorhabditis elegans*, *Anopheles gambiae*, and *D. melanogaster*, which have demonstrated that the sexes differ substantially in the genes they express [21-23].

**Figure 4:**
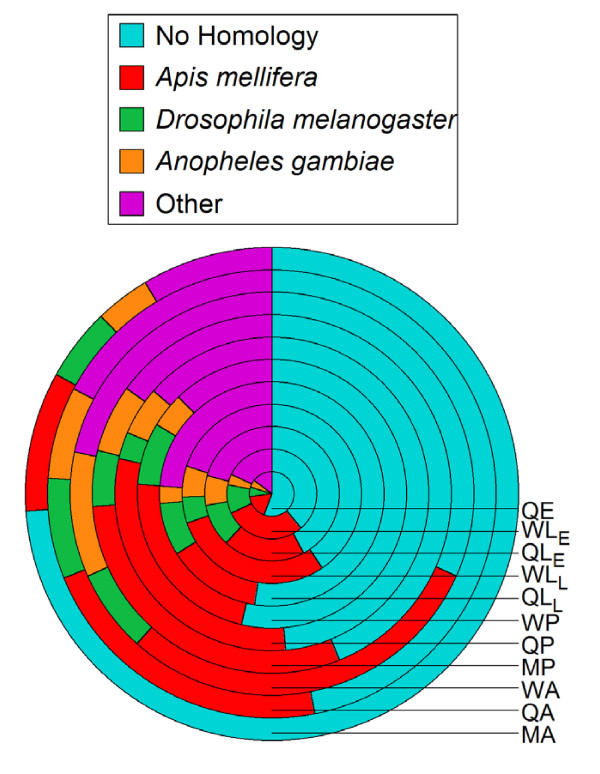
Distribution of GenBank Blast matches (expectation (e) values < 10^-5^) within each *V. squamosa *cDNA library. Q, queen; W, worker; M, male; E, egg; L_E_, early larval instars; L_L_, late larval instars; P, pupa; A, adult.

Our analysis of sequenced ESTs uncovered another interesting pattern regarding sex in *V. squamosa*. We found that the proportion of ESTs matching known sequences in GenBank varied significantly among libraries (G_10 _= 102.60; *p *< 0.0001). Specifically, the differences in the proportion of genes displaying homology in the adult male and adult female libraries is striking (Figure 4), with adult females exhibiting significantly higher (G_1 _= 25.51, *p *< 0.001) numbers of homologs (adult workers 69%, adult queens 53%) compared with adult males (26%). One possible explanation for these differences is that genes expressed in adult males evolve particularly rapidly relative to those expressed in females and at other developmental stages. This result is consistent with studies in other taxa that have shown that male-specific genes evolve rapidly [24-26]. Indeed, Singh and Kulathinal [27] deduced from comparative analyses of genome evolution that much *de novo *gene evolution occurs among male-biased genes. Our data indicate that similar processes may operate in social insects. Whether such putatively rapidly evolving genes are exclusively or primarily expressed in sex-specific tissues, as has been found to be the case in other taxa [24], represents an area of future research. Regardless, our suggestion that genes expressed in males may evolve differently from those expressed in females is notable because it points to the importance of males in the evolution of social-insect populations, a subject that until recently has been largely ignored [28].

### Expression patterns and development of caste

The defining feature of social insects is the division of individuals into reproductive and sterile castes [29]. Therefore, considerable research has focused on identifying genes that are differentially expressed between castes. For example, the molecular basis of caste differences has been investigated in bees [30-35], ants [36,37], wasps [38], and termites [13,39-41]. Overall, these studies represent at least five independent evolutionary events leading to sociality (summarized by Sumner et al [38]).

How does caste development in *V. squamosa *compare with these other taxa? Four major trends arise from our analyses. First, as might be expected, EST chord distances among castes at earlier life stages are more similar (*WL*_*E*_-*QL*_*E *_= 1.08) than at later life stages (*WA*-*QA *distance = 1.23), with intermediate stages at intermediate distances (see Table 1 for library definitions). This result indicates that as castes diverge phenotypically, physiologically, and behaviorally, patterns of gene expression also become increasingly divergent.

Second, our data contrast with a pattern of development identified in honeybees. Evans and Wheeler [31] suggested that patterns of expression of worker-destined larvae and younger bipotent larvae were more similar than between queen-destined larvae and bipotent larvae in *A. mellifera*. However, in *V. squamosa*, we found the opposite pattern when either early worker larvae (chord distances: *WL*_*E*_-*WL*_*L *_= 1.28, *WL*_*E*_-*QL*_*L *_= 1.21) or early queen larvae (chord distances: *QL*_*E*_-*WL*_*L *_= 1.29, *QL*_*E*_-*QL*_*L *_= 1.16) were used in the comparison. Thus, the trends in our data indicate that young larvae are more similar to queen-destined larvae than to worker-destined larvae in *V. squamosa*. Additionally, the observed differences in chord distances between worker or queen early larvae and worker or queen late larvae suggest that even though young larvae (i.e., *WL*_*E *_and *QL*_*E*_) are potentially bipotent, they may express different genes.

Third, despite the differences in overall patterns of caste differentiation mentioned above, some patterns of gene function are conserved. Specifically, early queen larvae of *V. squamosa *express more genes related to metabolism (*G*_1 _= 4.70, *p *< 0.05) than do similarly aged worker larvae (Figure 3). A similar pattern has been found in both the wasp *P. canadensis *[38] and the bee *A. mellifera *[31]. It is unclear why genes associated with metabolism show increased expression only in certain stages of queen development. It is possible that overexpression of metabolic genes early in ontogeny is sufficient to spur rapid growth in *Apis *queens, which develop faster than *Apis *workers. Similarly, *Vespula *queens are fed more than *Vespula *workers in the early larval instars [42], which may be a consequence of higher metabolic rates at these early stages and may ultimately lead to the large size differences observed between the castes. Regardless, queen production seems to be associated with increased energy production in hymenopteran social insects. This implies that gene function related to caste development may be conserved.

Fourth, the hexamerin gene family, which plays a significant role in caste differentiation in *A. mellifera *[30], *B. terrestris *[33] and *R. flavipes *[13,40,41], also shows significant differential expression in *V. squamosa *(VSQ019, VSQ232, VSQ233, VSQ292; Table 1). The arylphorin-like hexamerin most highly expressed in *V. squamosa *(VSQ019) is a methionine-rich member of the hexamerin family that participates in the storage of amino acids accumulated during larval development [43]. Moreover, as is the case in other social insect taxa, the different hexamerin ESTs in *V. squamosa *exhibit different expression patterns between queen and worker castes. VSQ019 and VSQ233 are upregulated in queen-destined larvae of *V. squamosa*, whereas VSQ232 and VSQ292 (both hexamerin 70b-like ESTs) are more highly expressed in the late larvae of workers relative to queens. The similarity of this gene-expression pattern among these species suggests that some specific pathways are conserved during social-insect evolution.

## Conclusion

We conducted the first study to simultaneously address the influence of developmental stage, sex, and caste on patterns of gene expression. We found that patterns of expression are more similar across castes for a specific developmental stage than within castes at different stages. Similar to other insect taxa, larvae of our study taxon *V. squamosa *upregulate genes related to metabolism and possessing structural activity. Furthermore, our data provide a provocative example of divergent selection pressures for genes expressed differentially between the sexes. We also discovered that *V. squamosa *and *A. mellifera *castes, which arose via independent evolutionary events, may develop through different trajectories. Nevertheless, at least a few specific gene functions and at least one specific gene family appear to be conserved components of caste differentiation. Overall, our results illustrate how the study of phenotypic diversity arising from patterns of gene expression can illuminate evolutionary effects of development in animal taxa.

## Methods

### cDNA library construction, processing and assembling

We constructed 11 directional cDNA libraries from several developmental stages of the wasp *V. squamosa*. The 11 libraries contained copies of transcripts obtained from: (i) eggs collected from queen cells; pooled female larvae from the first three early larval instars sampled from (ii) queen cells and (iii) worker cells; pooled female larvae of the fourth and fifth late larval instars sampled from (iv) queen cells and (v) worker cells; (vi) queen pupae; (vii) worker pupae; (viii) male pupae; (ix) queen adults; (x) worker adults; and (xi) male adults. The cDNA libraries were synthesized using a commercial construction kit (pBluescript^® ^II XR cDNA Library Construction Kit; Stratagene, La Jolla, CA, USA). In total, 4224 independent clones were isolated from these libraries, and 3388 single-pass sequences were obtained using the SK primer. After cloned sequences were filtered for vector contamination and quality, we obtained 2144 expressed sequence tags (ESTs; GenBank accession numbers: EG325041–EG327184).

### EST processing and assembling

These ESTs were grouped into clusters using the BLASTN algorithm [44]. When sequences from all 11 libraries were analyzed in parallel, 760 sequences were unique, and the remaining sequences formed 294 clusters of two or more sequences, giving a total of 1054 unigenes (Table 2). Within each library, the mean ± SE number of ESTs was 194 ± 9.2 (range 147–233), and the frequency of private ESTs was 0.38 ± 0.049 (Table 2) (range 16.8–66.3). Furthermore, the gene diversity [45] for each library, which represents the probability of drawing two distinct sequences from a library by chance, ranged from 0.970 to 0.994, indicating that the libraries contained many unique sequences (Table 2). BLASTX similarity searches [44] indicated that 52% of all the ESTs showed similarity to known sequences (e<10-^5^; Figure 4), a frequency not substantially different from previous studies in other Hymenoptera [20,46].

### Digital gene-expression analysis

We clustered the 11 libraries using the neighbor-joining method based on chord distances derived from library EST frequencies in order to gain an understanding of how patterns of gene expression were associated with development [47]. Furthermore, we explored variation in the genes expressed among libraries using digital methods [48]. This approach uses large-scale non-normalized random 3' -end cDNA library sequencing [49], but is extensible to any methodical sequencing strategy. The level of expression within each tissue is estimated from the number of cognate ESTs found in each library, under the assumption that it is proportional to the transcript frequencies [50,51]. These tests were conducted with the software program IDEG6 [52]. Overall, these methods may not provide accurate estimates of the absolute frequencies of particular genes, if certain gene sequences are subject to cloning biases. In addition, these techniques are unlikely to detect genes expressed at low levels, such as those with regulatory functions. Nevertheless, this approach can be reliably used to detect genes differentially expressed among libraries.

## Authors' contributions

EH and MG conceived the study and participated in its design and coordination. EH carried out the library construction, DNA sequencing, and EST analysis. EH and MG outlined the manuscript together and EH drafted the manuscript. Both authors have read and approved the final manuscript

## Figures and Tables

**Table 1:** Expression levels for 52 genes present at significantly different frequencies (*p *< 0.01) across cDNA libraries. Identity of putative homologous sequence and expectation (E) value of each match are presented. Raw number of sequences found in each library and the *p *value indicating significant differentiation of ESTs are also provided.

Gene ID	GenBank ID	Homolog	e-value	*QE*	*WL*_*E*_	*QL*_*E*_	*WL*_*L*_	*QL*_*L*_	*WP*	*QP*	*MP*	*WA*	*QA*	*MA*	*p*
VSQ001	EG326522	*Ctenocephalides felis *serpin	1e-021	0	0	0	3	0	0	0	0	1	0	0	0.00239
VSQ005	EG326963	*Dermatobia hominis *NADH dehydrogenase subunit 1	2e-034	0	0	0	3	3	0	0	0	0	1	0	0.00755
VSQ018	EG326808	*Blattella germanica *similar to major allergen Bla g 1.02	5e-035	0	3	2	7	22	0	0	0	0	0	0	<0.0001
VSQ019	EG326728	*Apis mellifera *similar to arylphorin-like hexamerin	1e-18	1	0	0	11	31	0	0	0	0	0	1	<0.0001
VSQ020	EG326434	*Apis mellifera *similar to glutamate receptor IB	6e-039	0	0	0	2	0	0	0	0	0	0	0	0.00794
VSQ031	EG326500	*Polistes dominulus *odorant-binding protein OBP-1 precursor	8e-029	0	0	0	1	0	2	0	0	6	3	0	0.00226
VSQ040	EG325267	*Tetraodon nigroviridis *unnamed protein product	3e-08	0	0	0	0	0	0	0	0	5	0	0	<0.0001
VSQ048	EG326323	Human herpesvirus 6 U88	1e-28	0	1	5	0	2	0	0	0	0	0	0	0.00123
VSQ050	EG326528	*Drosophila melanogaster *TPA: HDC07203	8e-12	1	9	8	0	2	0	1	0	3	1	0	<0.0001
VSQ051	EG325164	No homology		0	5	15	0	2	0	0	0	0	0	0	<0.0001
VSQ056	EG326606	No homology		2	1	4	0	22	0	0	0	0	0	0	<0.0001
VSQ058	EG326432	No homology		0	16	4	3	7	0	0	1	0	0	2	<0.0001
VSQ074	EG327162	*Apis mellifera *similar to ribosomal protein L35A	3e-55	0	0	4	0	0	0	1	1	0	0	0	0.00935
VSQ089	EG326517	No homology		0	3	1	0	1	0	0	0	0	0	0	0.00662
VSQ096	EG325485	*Drosophila melanogaster *RNA polymerase II accessory factor rpb4	5e-47	0	0	3	0	0	0	0	0	0	0	0	0.00364
VSQ122	EG326342	*Apis mellifera *similar to ENSANGP00000015316	2e-29	0	2	16	0	3	0	0	0	0	0	0	<0.0001
VSQ156	EG326487	*Anopheles gambiae *ENSANGP00000014145	5e-13	0	4	1	0	0	0	0	0	0	0	0	<0.0001
VSQ169	EG326695	*Drosophila pseudoobscura *GA11614-PA	7e-70	0	0	1	1	5	0	0	0	1	0	0	0.00296
VSQ173	EG326465	*Apis mellifera *similar to glucose dehydrogenase	7e-67	0	2	0	0	0	0	0	0	0	0	0	0.00337
VSQ176	EG326537	*Apis mellifera *similar to ribosomal protein S5	1e-90	0	3	0	0	0	0	0	0	0	0	0	<0.0001
VSQ185	EG326415	*Ixodes scapularis *beta-adaptin	1e-41	0	2	0	0	0	0	0	0	0	0	0	0.00337
VSQ211	EG326474	No homology		0	2	0	0	0	0	0	0	0	0	0	0.00337
VSQ212	EG326484	*Anopheles gambiae *ENSANGP00000023258	6e-07	0	2	0	0	0	0	0	0	0	0	0	0.00337
VSQ232	EG326810	*Apis mellifera *hexamerin 70b	6e-094	0	0	0	3	2	0	0	0	0	0	0	0.0035
VSQ233	EG326613	*Camponotus festinates *hexamerin 2	4e-054	0	0	0	0	3	0	0	0	0	0	0	0.00314
VSQ249	EG326612	No homology		0	0	0	2	4	0	0	1	0	0	0	0.00709
VSQ254	EG325941	*Apis mellifera *short-chain dehydrogenase/reductase	3e-41	0	0	0	0	2	1	5	0	0	0	0	<0.0001
VSQ280	EG326741	*Apis mellifera *similar to apontic CG5393-PB, isoform B	6e-21	0	0	0	0	1	0	0	0	0	3	0	0.00481
VSQ292	EG326813	*Apis mellifera *hexamerin 70b	9e-32	0	0	0	3	1	0	0	0	0	0	0	0.0021
VSQ296	EG326805	*Vespula vulgaris *cytochrome b	1e-067	0	0	0	3	0	0	0	0	0	0	1	0.00163
VSQ303	EG326821	*Lysiphlebus testaceipes *ribosomal protein L3 variant 1	3e-68	0	0	0	2	0	0	0	0	0	0	0	0.00794
VSQ307	EG326924	*Apis mellifera *similar to eukaryotic initiation factor 4A	6e-74	0	0	0	2	0	0	0	0	0	0	0	0.00794
VSQ318	EG326264	*Drosophila yakuba *odorant-binding protein 56e	8e-15	2	0	0	1	0	7	4	6	1	1	2	0.00158
VSQ338	EG325431	*Apis mellifera *similar to bellwether CG3612-PA isoform 1	e-105	0	0	0	1	0	0	0	0	4	0	0	0.00648
VSQ349	EG325944	*Tenebrio molitor *86 kDa early-staged encapsulation inducing protein	5e-14	0	0	0	1	0	0	3	0	0	0	0	0.00326
VSQ352	EG326946	No homology		0	0	0	2	0	0	0	0	0	0	0	0.00794
VSQ363	EG326968	No homology		0	0	0	0	0	0	0	0	0	0	6	<0.0001
VSQ378	EG326993	No homology		0	0	0	0	0	0	0	0	0	0	3	0.00014
VSQ389	EG327083	No homology		0	0	0	0	0	0	0	0	0	0	9	<0.0001
VSQ437	EG327159	*Vespa crabro *chemosensory protein	4e-61	0	0	0	0	0	0	0	0	0	3	1	0.00376
VSQ445	EG325384	*Periplaneta americana *Cr-PII allergen	8e-16	0	0	0	0	0	0	0	0	5	1	1	0.00311
VSQ463	EG326174	No homology		0	0	0	0	0	4	4	0	0	1	0	<0.0001
VSQ501	EG325247	*Apis mellifera *similar to CG14934-PA	2e-77	0	0	0	0	0	0	0	0	5	1	0	0.00059
VSQ565	EG325238	No homology		0	0	0	0	0	0	0	0	4	0	0	0.00101
VSQ581	EG325293	*Apis mellifera *similar to kazal-type proteinase inhibitor	9e-026	0	0	0	0	0	0	0	1	4	0	0	0.00847
VSQ682	EG325660	*Lysiphlebus testaceipes *ribosomal protein L3 variant 1	2e-76	7	0	0	0	0	0	0	0	0	0	0	0
VSQ709	EG325659	*Pisum sativum *putative senescence-associated protein	4e-15	3	0	0	0	0	0	0	0	0	0	0	0.00066
VSQ803	EG325675	*Anopheles gambiae *ENSANGP00000011747	9e-33	0	0	0	0	0	0	0	3	0	0	0	0.00677
VSQ850	EG326242	No homology		0	0	0	0	0	6	0	2	0	0	0	0
VSQ943	EG326146	*Human coxsackievirus *polyprotein	4e-05	0	0	0	0	0	17	29	0	0	0	0	0
VSQ954	EG326048	No homology		0	0	0	0	0	2	4	0	0	0	0	0.00016
VSQ955	EG325987	*Apis mellifera *hypothetical protein XP_397583	3e-12	0	0	0	0	0	1	3	0	0	0	0	0.00346

EST, expressed sequence tag.

Q, queen; W, worker; M, male; E, egg; L_E_, early larval instars; L_L_, late larval instars; P, pupa; A, adult.

**Table 2:** Numbers and clustering of ESTs among different *V. squamosa *libraries. n corresponds to the total number of ESTs sequenced per library. Contigs are ESTs represented multiple times while Singletons are ESTs present only once per library. Unigenes equal the total number of different ESTs in a library. Private EST's are sequences that occur only in that specific library. Gene diversity is the probability of drawing two distinct sequences from a library by chance.

Library	*QE*	*WL*_*E*_	*QL*_*E*_	*WL*_*L*_	*QL*_*L*_	*WP*	*QP*	*MP*	*WA*	*QA*	*MA*	Total
*n*	196	147	208	145	231	172	177	233	222	196	206	2133
Contigs	15	26	37	24	35	19	25	26	34	27	18	294
Singletons	151	68	94	94	61	108	83	170	158	116	112	760
Unigenes	166	94	131	118	96	127	108	196	192	143	130	1054
Private ESTs	114	37	50	53	33	65	46	135	120	82	88	823
Gene diversity	0.998	0.982	0.986	0.991	0.957	0.987	0.970	0.998	0.997	0.986	0.994	

EST, expressed sequence tag.

Q, queen; W, worker; M, male; E, egg; L_E_, early larval instars; L_L_, late larval instars; P, pupa; A, adult
